# 5-Fluorouracil Nanoparticles Inhibit Hepatocellular Carcinoma via Activation of the p53 Pathway in the Orthotopic Transplant Mouse Model

**DOI:** 10.1371/journal.pone.0047115

**Published:** 2012-10-15

**Authors:** Mingrong Cheng, Bing He, Tao Wan, Weiping Zhu, Jiang Han, Bingbing Zha, Houxiang Chen, Fengxiao Yang, Qing Li, Wei Wang, Hongzhi Xu, Tao Ye

**Affiliations:** 1 Department of General Surgery, Zhoupu Hospital of Shanghai Pudong New Area, Shanghai, China; 2 Department of General Surgery, Shanghai Fifth People’s Hospital, Fudan University, Shanghai, China; 3 Biomedical Materials and Engineering Center, Wuhan University of Technology, Wuhan, China; 4 Department of Endocrine, Shanghai Fifth People’s Hospital, Fudan University, Shanghai, China; 5 Department of General Medicine, Pujiang Hospital of Shanghai Fifth People’s Hospital, Shanghai, China; Virginia Commonwealth University, United States of America

## Abstract

Biodegradable polymer nanoparticle drug delivery systems provide targeted drug delivery, improved pharmacokinetic and biodistribution, enhanced drug stability and fewer side effects. These drug delivery systems are widely used for delivering cytotoxic agents. In the present study, we synthesized GC/5-FU nanoparticles by combining galactosylated chitosan (GC) material with 5-FU, and tested its effect on liver cancer *in vitro* and *in vivo*. The in vitro anti-cancer effects of this sustained release system were both dose- and time-dependent, and demonstrated higher cytotoxicity against hepatic cancer cells than against other cell types. The distribution of GC/5-FU in vivo revealed the greatest accumulation in hepatic cancer tissues. GC/5-FU significantly inhibited tumor growth in an orthotropic liver cancer mouse model, resulting in a significant reduction in tumor weight and increased survival time in comparison to 5-FU alone. Flow cytometry and TUNEL assays in hepatic cancer cells showed that GC/5-FU was associated with higher rates of G0–G1 arrest and apoptosis than 5-FU. Analysis of apoptosis pathways indicated that GC/5-FU upregulates p53 expression at both protein and mRNA levels. This in turn lowers Bcl-2/Bax expression resulting in mitochondrial release of cytochrome C into the cytosol with subsequent caspase-3 activation. Upregulation of caspase-3 expression decreased poly ADP-ribose polymerase 1 (PARP-1) at mRNA and protein levels, further promoting apoptosis. These findings indicate that sustained release of GC/5-FU nanoparticles are more effective at targeting hepatic cancer cells than 5-FU monotherapy in the mouse orthotropic liver cancer mouse model.

## Introduction

Cancer is a leading cause of death worldwide. It has been estimated that there will be 12 million cancer deaths worldwide in 2030 [1]. Liver cancer is the fifth most frequently diagnosed cancer and the third leading cause of cancer: related death in the world [2]. This aggressive disease is a major global threat to public health, with an estimated 748,300 new cases and 695,900 cancer deaths occurring worldwide in 2008. Most of these cases occur in developing countries and half of them in China alone [3]. More than 50% of patients presenting with earlier stages of the disease undergo surgical resection, but even after curative resection, 80% of patients develop new tumors in the residual liver tissue within 2 years and eventually die due to progressive disease [4].

Chemotherapy is the main treatment for advanced or recurrent liver cancer [5].

5-Fluorouracil (5-FU) is a cytotoxic drug, which interferes with nucleic acid synthesis, inhibits DNA synthesis, and eventually halts cell growth [6]. It is extensively used to treat solid tumors such as liver, breast, colorectum and brain cancer. However, 5-FU is rapidly metabolized, which means administration by intravenous injection or infusion is required to maintain therapeutic blood levels. In common with most chemotherapy drugs, 5-FU is associated wide ranging side effects among which severe gastrointestinal toxicity, hematologic disturbance, and severe bone marrow deficiency, remain significant limitations to its clinical use [7], [8].

Drug delivery systems which provide controlled release of therapeutic drug doses directly to the site of tumor have been tested as an alternative approach to systemic drug administration [9], [10]. These systems provide extended and continuous localized drug delivery resulting in high drug concentrations within the tumor microenvironment and low drug concentrations in the blood stream and other organs [11], [12]. The therapeutic index of 5-FU is narrow and its half life of 5-FU in blood and body tissues is very short, in the region of minutes. Site-specific delivery of 5-FU would reduce the systemic side effects and provide effective and safe therapy.

Nanoparticles have been shown to be delivered to specific sites by size-dependant passive targeting [13], [14]. Passive delivery occurs when nanoparticles are transported through leaky tumor capillary fenestrations into the tumor cells by diffusion or convection [15]. Active targeting involves the coupling the target-specific molecules, such as ligands, binding sites, or antibodies, to the surface of the nanoparticles to assist drug distribution to specific target tissues [16]. Targeted nanoparticle drug carrier technology is characterized by high selectivity, clinically significant therapeutic effect, low toxicity in normal tissues, and low interaction potential with associated drugs. In vivo experiments show that nanoparticle drug carrier systems are not associated with significant side effects because of their ability to bind to chemotherapeutic drugs and efficiently deliver them to various cell types [20], [21], [22]. This form of targeted therapy, therefore, provides an ideal mechanism for improving the clinical effectiveness and bioavailability of a therapeutic product and for reducing the side effects associated with agents currently used in liver cancer [17], [18], [19].

In recent years, much attention has been focused on using natural and synthetic polymers as nanoparticles, due to their good biocompatibility, biodegradability and ability to prolong drug release [23], [24]. Chitosan (CS) is a natural linear biopolyaminosaccharide derived from alkaline deacetylation of chitin. It has been found to be the second most abundant biopolymer in nature behind only cellulose. Galactosylated chitosan (GC) is a galactose ligand, with a chitosan modified molecular structure [25]. Asialoglycoprotein receptor (ASGPR) is found on membranes of hepatocytes that face sinusoids. Each hepatocyte contains approximately two million binding sites for ASGPR [26]. This ASGPR receptor shows specificity for glycoproteins which contain exposed galactose or acetyl galactosamine groups. The binding of the galactose ligand with ASGPR has been shown to induce liver-targeted gene transfer.

We have previously synthesized a GC nanoparticle which successfully transferred genes into the liver in vitro and in vivo. We also confirmed that this nanoparticle material was a high selectivity for the liver and was associated with low cytotoxicity [27]. In the present study, we synthesized GC/5-FU nanoparticles by combining the GC material with 5-FU, and tested its effect on liver cancer in vitro and in vivo. We found that the GC/5-FU nanoparticles specifically targeted the liver and that the addition of GC increases the cytotoxicity of 5-FU.

**Figure 1:**
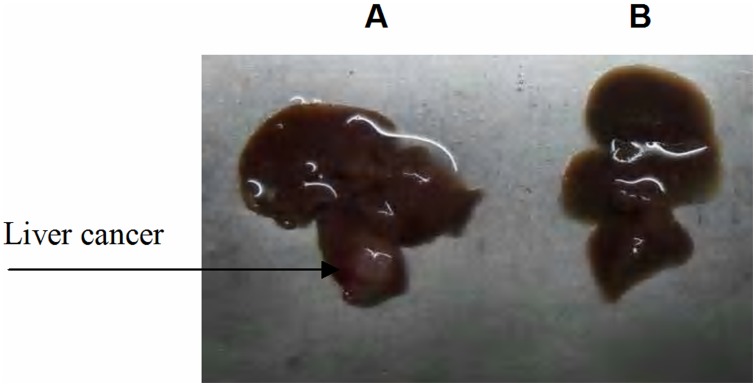
Establishment of the hepatic cancer mouse model. (A) Liver cancer; (B) Normal mouse liver.

## Materials and Methods

### Reagents

Chitosan (CS, with >85% deacetylation), 1-ethyl-3-(3-dimethylaminopropyl) carbodiimide hydrochloride (EDC), N-hydroxysuccinimide (NHS) and RNase were purchased from Sigma, USA. GC was synthesized and stored by our group. HCl was obtained from the Shanghai Medpep, AR. LC-10A HPLC system (Shimadzu, Japan), and fluorescein isothiocyanate (FITC) was obtained from Sigma-Aldrich (Saint Louis, MO, USA) and the flow cytometry kit was obtained from (FACS CALIBUNR, USA). The TUNEL assay kit was obtained from Roche, Germany and the immunohistochemistry kit from GBI, USA. Caspase-3 and poly ADP-ribose polymerase 1 (PARP-1) antibodies were provided by Santa Cruz, CA, USA; Bax and Bcl-2 antibodies were provided by Temecula, CA, USA; and p53 antibody was provided by Beverly, MA, USA.

**Figure 2:**
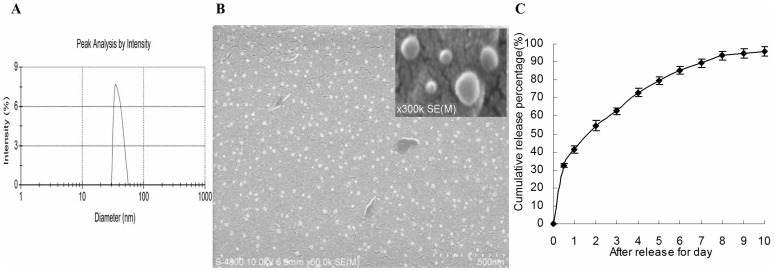
Particle size and scanning electron microscope (SEM) image of GC/5-FU. (A) Particle size graph showing the diameter of GC/5-FU (35.19±9.50 nm). (B) SEM image of GC/5-FU. The particles show spherical structure with a smooth surface and no adhesion between nanoparticles. (C) The in vitro release curve of nanoparticles in simulated body fluid (37°C, pH 7.4). A rapid release was observed from 0 h to 12 h, with a cumulative release percentage of 32.4%. Smooth slow-release occurred between Day 1 and 8, with a cumulative release percentage of 93.50%. During Day 8 to 10, the release reached a plateau, with a cumulative release percentage of 95.70% at Day 10.

### Mice and Cell Lines

The human hepatocellular carcinoma cell line (SMMC-7721) and normal liver cells (LO2) were obtained from the Committee on Type Culture Collection of Chinese Academy of Sciences (Shanghai, China). The human colon cancer cell line (SW480) was purchased from the American Type Culture Collection (Manassas, VA,USA), and the mouse hepatoma cell line H22 was purchased from the China Center for Type Culture Collection (CCTCC, Wuhan, China). Female BALB/c mice, 7 weeks of age and weighing about 20 g, were obtained from the Science Department of Experimental Animals of Fudan University in China. All mice were housed in a SPF level B animal facility. The study was approved by the Review Board of Shanghai Zhoupu Hospital and Fudan Medical College.

**Figure 3:**
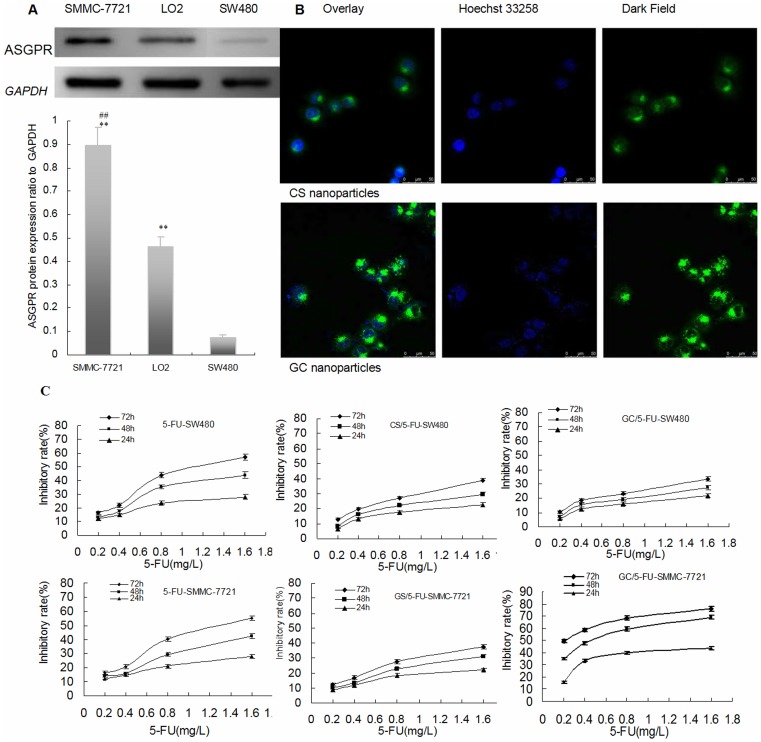
5-FU nanoparticle enhanced 5-FU to inhibit the growth of hepatic caner through ASGPR -mediated endocytosis *in vitro*. (A) ASGPR protein expression was detected by western blot in SMMC-7721, SW480 and LO2 cell lines (n = 3). The ASGPR protein expression in SMMC-7721 and LO2 was stronger than with SW480 (P<0.01), the expression in SMMC-7721 increased significantly than those with SW480 (P<0.01). **P<0.01 compared to SW480, ^##^P<0.01; compared to LO2. (B) Concurrent focal images of SMMC-7721 cells after 4 h incubation with CS and GC nanoparticles. The strong green fluorescent bright spots in SMMC-7721 cells were observed in the GC nanoparticles using a laser scanning confocal microscope and only small amount of green fluorescent spots were found in the CS nanoparticles without galactosyl ligand modified chitosan. So it was confirmed that the GC nanoparticles targeted liver cancer cells and entered into cells via ASGPR-mediated endocytosis. (C) Comparison of the inhibition rates of 5-FU, CS/5-FU and GC/5-FU on SW480 and SMMC-7721 cells. Results are shown as a average ± means and standard deviation (n = 3). At the 24, 48 and 72 h time points, the rate of tumor inhibition rate decreased from in the order GC/5-FU-SMMC-7721 to 5-FU-SW480 to 5-FU-SMMC-7721 to CS/5-FU-SMMC-7721 to CS/5-FU-SW480 to GC/5-FU-SW480.

### Synthesis of GC/5-FU

The 5-FU/GC was mixed for 30 s at a mass ratio of 10∶1 in solution, using a vortex oscillator (2500 rpm). The product was kept at room temperature for 30 min to assess further particle formation. Nanoparticles were purified by centrifugation at 10000 rpm for 10 min. The residues (including NaOH and 5-Fu) were washed in a large volume of de-ionized water, freeze-dried, and stored. The final concentration of 5-FU was 1.857 mg/mL. The product was stored at 4°C. The drug loading and encapsulation was calculated as the amount of 5-FU within the nanoparticles/nanoparticle mass × 100%. Encapsulation efficiency was calculated as the amount of 5-FU within the nanoparticles/total amount of 5-FU added × 100%.

### In vitro Release Experiment

Nanoparticles (20 mg) were mixed with 30 mL of simulated body fluid (SBF, pH 7.4) in dialysis bags and incubated at 37°C using a shaker with a fixed speed of 60 rpm. Samples were analyzed at 0.5, 1, 2, 3, 4, 5, 6, 7, 8, 9 and 10 days post-mixing. Optical density (OD) was measured at 265 nm using an automated microplate reader (Bio-Rad). The amount of 5-FU released at different time points was calculated according to a standard absorbance curve. The concentration and cumulative percentage release were calculated according to the standard curve equation. Each experiment was performed in triplicate.

**Table 1:** Comparison of IC_50_ of GC/5-FU, CS/5-FU and 5-FU for SW480 and SMMC-7721 cells (n = 3).

Group	The time of culture (day)									
	Day 1	Day 2	Day 3	Day 4	Day 5	Day 6	Day 7	Day 8	Day 9	Day 10
5-FU-SW480	2.102±0.167	1.643±0.034	1.234±0.031	0.943±0.033	0.752±0.046	0.649±0.054	0.633±0.042	0.622±0.042	0.612±0.042	0.607±0.043
CS/5-FU-SW480	2.354±0.191	1.895±0.054	1.604±0.034	1.105±0.047	0.769±0.048	0.632±0.047	0.446±0.051	0.388±0.038	0.358±0.045	0.276±0.035
GC/5-FU-SW480	2.432±0.272	2.034±0.043	1.768±0.038	1.313±0.055	0.977±0.033	0.668±0.054	0.481±0.037	0.403±0.036	0.362±0.032	0.271±0.021
5-FU-SMMC-7721	2.248±0.263	1.853±0.052	1.549±0.034	1.103±0.028	0.900±0.014	0.809±0.030	0.742±0.031	0.694±0.042	0.690±0.042	0.683±0.054
CS/5-FU-SMMC-7721	2.418±0.192	2.046±0.045	1.625±0.046	1.206±0.058	0.912±0.046	0.765±0.028	0.578±0.029	0.456±0.035	0.414±0.034	0.375±0.034
GC/5-FU-SMMC-7721	2.017±0.186	1.079±0.032	0.784±0.042	0.632±0.067	0.523±0.054	0.364±0.032	0.256±0.021	0.222±0.012	0.167±0.015	0.114±0.012

### Cell Imaging and Staining Procedure

SMMC-7721 cells, SW480 cells and LO2 cells were plated on round cover slips and mounted onto 6-well plates for 24 h. The culture medium was replaced with fresh medium containing various FITC-labeled nanoparticles at a concentration of 0.1 mg/mL for 4 h. The cells were fixed with 4% paraformldehyde at room temperature for 20 min. The cell nuclei were stained with Hoechst 33258 solution, washed three times with 0.01 M PBS and mounted in glycerol buffer. Fuorescence images were observed by using Nikon A1 confocal laser scanning microscope (Nikon, Tokyo, Japan) at an excitation wavelength of 488 nm for FITC-labeled nanoparticles, and of 405 nm for Hoechst 33258. The images were superimposed using NIS Elements imaging software (Nikon, Tokyo, Japan).

**Figure 4:**
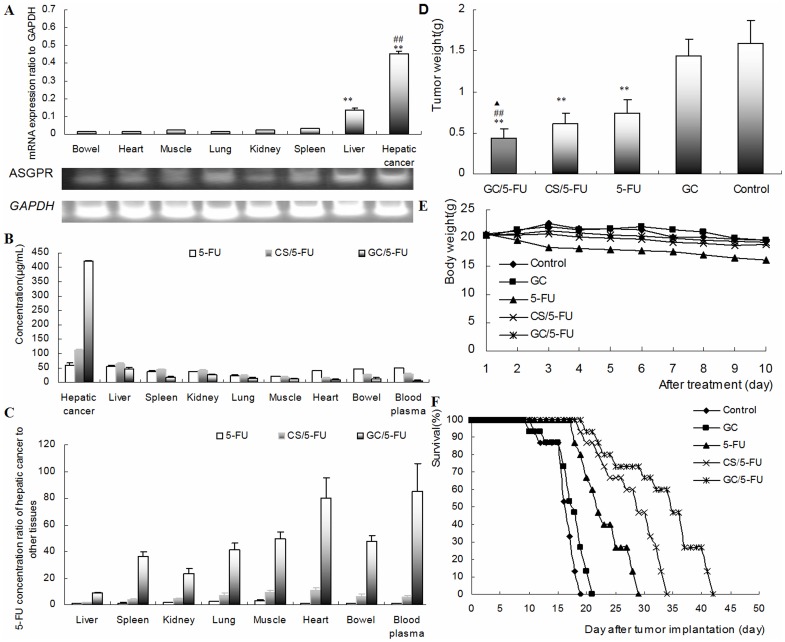
5-FU nanoparticle had liver-targeting and enhanced 5-FU to inhibit the growth of hepatic caner through ASGPR *in vivo*. (A) Expression of ASGPR mRNA in different tissues of hepatic cancer, liver, spleen, kidney, lung, muscle, heart and bowel in the orthotropic liver cancer mouse model evaluated by RT-PCR. Results are shown as means±SD (n = 3). (^**^P<0.01, compared with spleen, kidney, lung, muscle, heart and bowel; ^##^ P<0.01, compared with liver). (B) 5-FU concentration in different tissues in mice were treated with i.v. 5-FU, CS/5-FU or GC/5-FU or, 5-FU determined 30 min post-injection. Results are shown as an means±SD (n = 3). Hepatic cancer tissue showed the highest 5-FU concentration, followed by liver tissue. (C) The ratio of 5-FU concentration in hepatic cancer cells relative to that in other tissues. The 5-FU concentration in hepatic cancer was 8.69-, 23.35-, 79.96- and 85.15-fold higher than that in normal liver tissue, kidney, heart and blood, respectively. (D) The weight of liver tumors measured on Day 10. Results are shown as means±SD deviation (n = 10). Mice received GC/5-FU,CS/5-FU, 5-FU, GC or PBS on Day 5 after establishing the transplant model. **P<0.01 compared to control or GC group; ^##^P<0.01 compared to 5-FU group, ^▴^P<0.05 compared to CS/5-FU group. (E)Body weight was monitored from Day 1 to 10 of treatment (n = 10). Body weigh decreased significantly more in the 5-FU group compared with the control, GC, CS/5-FU and GC/5-FU group. There was no difference in body weight between the GC/5-FU, CS/5-FU, GC and control groups. (F) Mouse survival. The median survival in the control, GC, 5-FU, CS/5-FU and GC/5-FU groups was 16, 17, 22, 29 and 35 days, respectively. The longest median survival time was seen in the GC/5-FU group.

### The Effects of GC/5-FU on Tumor Cell Growth and IC50

SW480 and SMMC-7721 cells were grown in PRMI 1640 (GIBCO) supplemented with 10% FBS (Hangzhou Evergreen Biological Engineering Materials Co, Ltd) and incubated at 37°C with 5% CO_2_. Cells (190 μL) in the exponential growth phase were seeded at a density of 1×10^4^/mL into 96-well plates. After incubation for 24 h, 10 μL of various concentrations of 5-FU, chitosan (CS)/5-FU and GC/5-FU were added to individual wells and incubated for 1, 2, 3, 4, 5, 6, 7, 8, 9 and 10 days. Thiazolyl blue tetrazolium bromide (30 μg) was added to each well at the end point, followed by incubation for 4 h. The cells were then dissolved in 200 μL DMSO for 10 min. Absorbance (A) at 490 nm was measured using a Bio-Rad automated microplate reader. All the experiments were performed in triplicate. The MTT assay was performed 24, 48 and 72 h after addition of 5-FU, CS/5-FU or GC/5-FU. Tumor growth cell inhibition rate was calculated as (A_490_ of control well – A_490_ of experimental well)/A_490_ of control well ×100%. The IC_50_ was calculated using the Bliss method [28], [29].

**Figure 5:**
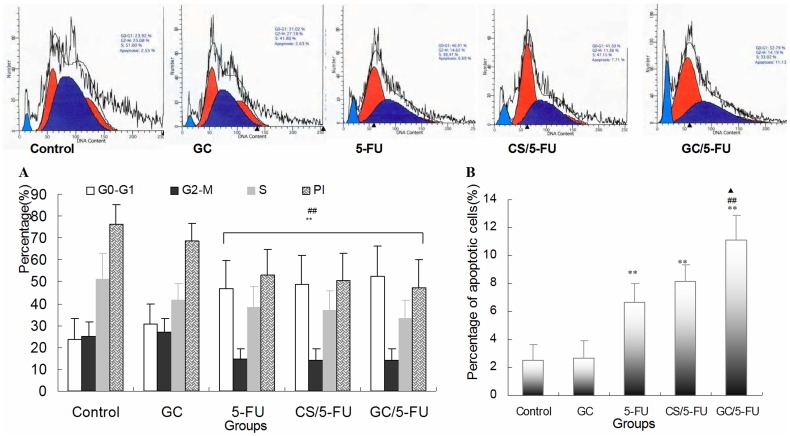
The effects of different treatments on cell cycle, proliferation index and apoptosis index by flow cytometry analysis of H22 cells. (A) Quantification of cell cycle distribution and proliferation index of H22 cells. Percentage of cells in G0–G1 in the GC/5-FU,CS/5-FU and 5-FU groups was higher than in the control and GC groups, and the proliferation index decreased significantly in the other three groups (P<0.01). (B) Quantification of apoptosis of H22 in different treatment groups. The percentage of apoptotic cells in the GC/5-FU,CS/5-FU and 5-FU groups was significantly higher than in the control and GC groups (P<0.01). The percentage of apoptotic cells in the GC/5-FU group was also higher than in the 5-FU (P<0.01) and CS/5-FU groups (P<0.05). **P<0.01 compared with control or GC group; ^##^P<0.01 compared with the 5-FU group; ^▴^P<0.05 compared with the CS/5-FU group.

**Figure 6:**
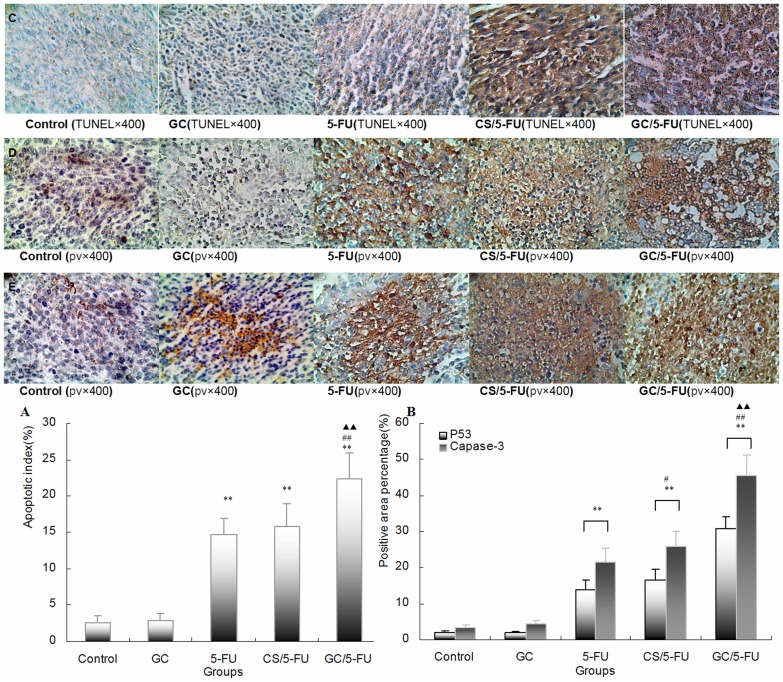
Apoptosis, p53 and caspase-3 expression were detected in tumor tissues in mouse model. (A) The quantification of the apoptotic index (AI) in hepatic cancer tissues. Apoptosis was detected using the TUNEL assay on the tumor sections. The tumor samples from the control and GC groups showed little apoptosis; the addition of 5-FU and CS/5-FU induced sporadic apoptosis, while GC/5-FU induced a high degree of apoptosis which showed a clustered distribution (C). The AI in the GC/5-FU, CS/5-FU and 5-FU groups was higher than that in the GC and control groups (P<0.01). The expression of AI in the GC/5-FU group increased more than in the CS/5-FU or 5-FU groups (P<0.01). (B) Quantification of p53 and caspase-3 expression detected by IHC. P53 (D) and Caspase-3 (E) staining in the control and GC groups showed a scattered cytoplasmic distribution pattern, appearing as dark yellow or dark brown. In the 5-FU, CS/5-FU and GC/5-FU groups, P53 and caspase-3 showed a sheet staining pattern, which was most marked in the GC/5-FU group. The expression of P53 and caspase-3 was higher in the 5-FU, CS/5-FU and GC/5-FU groups than in the control or GC groups. The highest increase was seen in the GC/5-FU group (P<0.01). **P<0.01 compared with control or GC group; ^##^P<0.01 compared with the 5-FU group; ^▴▴^P<0.01 compared with the CS/5-FU group.

### Animal Model

A subcutaneous liver cancer mouse model was established using the mouse hepatocellular cancer cell line H22. After euthanasia and dissection, fresh fast-growing tumor tissues were minced to form a tumor cell suspension at a density of 6×10^7^ cells/mL. Recipient mice were anesthetized with 20% urethane, followed by an injection of 50 µL of tumor cell suspension into the liver left lobe capsule. Approximately 2 min after completion of the procedure, when there was no leaking, the abdomen was sutured and the orthotropic liver cancer mouse model was established [30].

**Figure 7:**
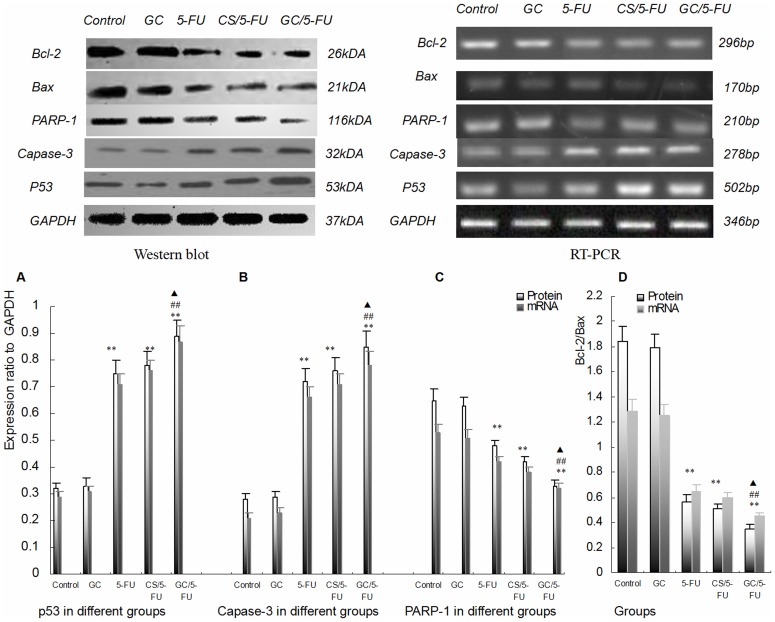
Expression of P53, caspase-3, Bax, Bcl-2 and PARP-1 in tumor tissues from mice. (A) Tissue levels of P53 were measured by RT-PCR and western blot, and normalized to GAPDH. The expression of P53 was higher in the 5-FU, CS/5-FU and GC/5-FU groups than in the control and GC groups. The highest expression was seen in the GC/5-FU group. (B) Caspase-3 expression in individual tumor samples was determined by both RT-PCR and western blot analysis and results were normalized to GAPDH. The expression of PARP-1 displayed a increasing tendency from control to GC to 5-FU to CS/5-FU to GC/5-FU groups (P<0.01), with the most significant increase being seen in the GC/5-FU group. (C) Expression of Bcl-2 and Bax was quantified by both RT-PCR and western blot. The ratio of Bcl-2/Bax showed a decreasing tendency from control to GC to 5-FU to CS/5-FU to GC/5-FU groups (P<0.01). Specifically, the ratio in 5-FU, CS/5-FU and GC/5-FU was significantly lower than in the control and GC groups. The lowest ratio was observed in the GC/5-FU group (P<0.01). (D) PARP-1 expression in individual tumor samples was determined by RT-PCR and western blot analysis; results were normalized to GAPDH. The expression of PARP-1 displayed a decreasing tendency from control to GC to 5-FU to CS/5-FU to GC/5-FU groups (P<0.01). The lowest expression was seen in the GC/5-FU group. **P<0.01 compared to control or GC group; ^##^P<0.01 compared to 5-FU group; ^▴^P<0.05 compared to CS/5-FU group.

### Determination of the Targeting Effect of GC/5-FU in vivo

#### Preparation of 5-FU standard curve

Standard solution was added to 0.5 mL of serum to produce a 5-FU solution at final concentrations of 0.1, 0.2, 0.5, 1.0, 5.0, 10 and 20 mg/L. Linear regression was used to produce a standard curve with 5-FU concentration as the x-axis and the measured 5-FU peak area as the y-axis.

#### The concentration of GC/5-FU in different tissues

After the tumor had been established in the mouse model, the mice (n = 15) were randomly assigned into three groups of five mice, receiving 5-FU, CS/5-FU or GC/5-FU at a volume of 200 µL (containing 0.371 mg 5-FU) administered via the tail veil. Tissues and organs including tumor mass, liver, spleen, kidney, lung, muscle, heart and small intestine were harvested 30 min post-injection. After washing with saline, 0.5 g to 1.0 g of tissue was homogenized and 0.5 mL serum was taken for determination of 5-FU concentration.

### Observation of the Curative Effect of GC/5-FU in the Orthotropic Liver Cancer Mouse Model

After 5 days the hepatic tumors had become established reaching a diameter of 4 mm to 6 mm (Figure 1). The mice with established tumors were randomly assigned to one of five groups. Mice in the control group received 200 µL saline by intravenous injection. Mice in the GC group received 200 µL of GC nanomaterial. In the other groups mice were injected with a 200 µL solution containing 0.371 mg 5-FU of either 5-FU, CS/5-FU or GC/5-FU, together with 200 µL GC and PBS in the GC and PBS groups. The drugs were given continuously for 5 days starting from Day 5 after establishment of the tumor. Ten mice were sacrificed on Day 15. Tumor growth and body weight were estimated in each group. The remaining 15 mice in each group were kept for survival analysis.

### Cell Cycle and Apoptosis Analysis by Flow Cytometry

A cell suspension was obtained using from 1 to 2 mm^3^ of tumor tissue from each group. Cells were washed three times in 0.1 mol/L PBS and fixed using 70% ethanol. They were then incubated in the dark with 50 mg/L PI, 1.0% Triton X-100 and 10 mg/L RNaseA for 30 min at 4°C. Apoptosis and cell cycle distribution were analyzed by flow cytometry. Proliferation index (PI) was calculated as (S+G_2_/M)/(G_0_/G_1_+S+G_2_/M).

### TUNEL Assay for Detection of in situ Apoptosis

Sections of liver tumor (4 µm) were fixed using 4% PFA, and digested with 0.02 mg/L proteinase K for 30 min at room temperature. The sections were soaked in 3% H_2_O_2_ for 5 min at room temperature to deactivate the endogenous peroxidases. They were then soaked in pH balance buffer for 20 min, and incubated at 37°C for 1.5 h with a solution containing 1∶20 terminal deoxynucleotidyl transferase (TDT) enzyme. The sections were washed, and incubated with antibody for 30 min at 37°C. Diaminobenzidine was used to develop the sections after a second round of washing. A negative control was obtained by using ddH_2_O instead of TDT.

At least 1000 cells from at least 10 scopes were counted using DMR+Q550 system (Laica) technology, and the apoptotic index (AI) was calculated. Apoptotic cells were recognized by the appearance of brown or tan stained nuclei [31], [32].

### Immunohistochemistry

The 4 µm liver tumor sections were deparaffinized by incubation at 65°C and soaked in 3% H_2_O_2_ for 10 min at room temperature to deactivate endogenous peroxidases. Antigen retrieval was performed using a microwave. The primary antibody was incubated at 37°C for 1 h in a humidified chamber and the secondary antibody was incubated at 37°C for 30 min. PBS was used instead of primary antibody for a negative control. The sections were washed, developed using DAB and counter stained with hematoxylin. The stained sections were allowed to dehydrate prior to being viewed under light microscopy [33]. Using this method, p53 staining was predominantly observed in the nucleus, which appeared brown and granular with a poorly defined background. Caspase-3 staining was mainly present in cytoplasm, which showed a brown granular staining pattern. The Image-pro plus 6.0 system was used to analyze five fields randomly chosen from each slide. The images were amplified 200-fold, converted into gray-scale so as to distinguish the positive staining area from background. The positive-stained area and the total area of the field were measured by the system. The area ratio was calculated as the staining area/total area ×100%. The stained area of each individual slide was determined by averaging the area ratio.

### RT-PCR

Primers were purchased from Shanghai R & S Biotechnology Co, Ltd. Tissue total RNA was extracted by TRIZOL (Invitrogen). Total RNA (1 μL) was reverse transcribed into cDNA using 0.5 μL AMV reverse transcriptase. PCR was performed using 2.5 μL cDNA, 0.1 μL Ex Taq HS, 0.1 μL forward primer and 0.1 μL reverse primer. The PCR reaction conditions were: 94°C for 2 min, 35 cycles of 94°C for 40 s, 50°C to 65°C for 40 s and 72°C for 1 min, followed by 72°C for 5 min. PCR products were kept in −20°C [34]. GAPDH was used as internal control. The PCR product (6 μL) was resolved in 2% agarose gel for 30 min (at 120 V, 100 mA) and stained with ethidium bromide solution for 5 min. The imaged was viewed using a UV gel imaging system, and analyzed using Quantity One software (Bio-Rad Inc). The expression of target genes was presented as the ratio of target to internal control GAPDH.

### Western Blot Analysis

Different concentrations of samples were loaded onto the 12% SDS-PAGE gel and resolved at 80 V followed by 120 V. Methanol-pretreated PVDF membranes were soaked in transfer buffer (pH 8.3, 25 mmol/L Tris-HCl, 192 mmol/L glycine, 20% methanol) for 10 min. Proteins on the SDS-PAGE gel were transferred onto the PVDF membrane and processed at 100 V for 70 min. The membranes were blocked by overnight exposure to 5% FBS/PBS at 4°C. Primary antibodies were diluted at 1∶2000 and incubated with the membranes for 3 min at room temperature. The membranes were then washed three times (10 min each time), with PBS containing 0.05% Tween 20. Goat-anti-mouse IgG secondary antibody (1∶8000) was added and incubated with the membrane for 3 h at room temperature. The membranes were then washed three times using the same washing solution, and developed for 1 min using an ECL kit with equal volumes of A and B solutions [35]. Image J version 1.44 software (National Institutes of Health) was used to analyze the average density values.

### Statistical Analysis

All data were expressed as means and standard deviations (±SD). Analysis of variance (ANOVA) was used to analyze within group data and one-way ANOVA was used to analyze between-group data. Least squares difference was used for pairwise comparisons between groups. Values of P<0.05 were considered statistically significant.

## Results

### Synthesis and Characterization of GC/5-FU Nanoparticles

5-FU/GC nanoparticles were successfully synthesized. The radius of the nanoparticles was normally distributed with a mean of 35.19±9.50 nm (Figure 2A). Electron microscopy showed that the particles had a regular spherical shape, with a smooth surface, uniform size, and there was no adhesion between nanoparticles (Figure 2B). The degree of drug loading was 6.12±1.36%, encapsulation efficiency was 81.82±5.32%, and the Zeta potential was +10.34±1.43 mV. Figure 2C shows the in vitro release curve of nanoparticles in simulated body fluid (37°C, pH 7.4). Rapid release was observed between 0 to 12 h, resulting in cumulative drug release of 32.4%, possibly due to the diffusion of surface 5-FU into the solution. This was followed by a period of smooth, slow release between Day 1 and 8, resulting in a cumulative release of 93.50%. Drug release plateaued between Days 8 and 10, with a cumulative release of 95.70% at Day 10.

### The Hepatocellular Cancer-specific Cytotoxicity of GC/5-FU Nanoparticles in Tumor Cells was Dose- and Time- dependent

Asialoglycoprotein protein (ASGPR) expression was detected by Western blot analysis in SMMC-7721, SW480 and LO2 cell lines (Figure 3A). ASGPR protein expression was more marked in SMMC-7721 and LO2 cells than in SW480 cells (P<0.01).

Strong green fluorescent bright spots in SMMC-7721 cells were observed in the GC nanoparticles after incubation for 4 h with FITC-labeled GC nanoparticles, whereas only small number of green fluorescent spots were found in FITC-labeled CS nanoparticles without galactosyl ligand modified chitosan. The GC nanoparticles targeted liver cancer cells and entered these cells through ASGPR -mediated endocytosis (Figure 3B).

As shown in Figure 3C, 5-FU, CS/5-FU and GC/5-FU inhibited the growth of SW480 and SMMC-7721 cells in a dose-dependent manner. At the 24, 48 and 72 h time points, the degree of tumor inhibition was maximal with GC/5-FU-SMMC-7721, and was progressively less marked with 5-FU-SW480, 5-FU-SMMC-7721, CS/5-FU-SMMC-7721, CS/5-FU-SW480, and GC/5-FU-SW480.

The time-dependency of cytotoxicity with GC/5-FU, CS/5-FU and 5-FU in SW480 and SMMC-7721 cells was compared by estimation of IC_50_ values during a 10 day period. As shown in Table 1, a time-dependent cytotoxic effect of 5-FU was seen during Day 1 to 6. During this period, the IC_50_ decreased. After 7 days, the cytotoxic effect of 5-FU-SW480 and 5-FU-SMMC-7721 reached a plateau. By contrast the time-dependent cytotoxic effect of GC/5-FU-SMMC-7721, CS/5-FU-SW480, CS/5-FU-SMMC-7721 and GC/5-FU-SW480 demonstrated an tendency to increase between Day 6 and 10. Thus, after Day 6, the IC_50_ values for CS/5-FU-SW480, CS/5-FU-SMMC-7721 and GC/5-FU-SW480 were lower than corresponding values for 5-FU-SW480 or 5-FU-SMMC-7721.

### Preparation of the 5-FU Standard Curve and Determination of in vivo Liver-targeting of GC/5-FU

The linear relationship between 5-FU (0.1 mg/L to 20 mg/L) and peak levels of 5-FU was defined by equation y = 2.5721x+0.6851 (r = 0.9956). The equation, was used to estimate the *in vivo* concentration of 5-FU in mice.

Concentrations of 5-FU were measured in the cancer tissue, liver, spleen, kidney, lung, muscle, heart, intestine and blood, 30 min after injection of 5-FU, CS/5-FU and GC/5-FU through tail vein of mice with orthotropic liver cancer. As shown in Figure 4A, the levels of ASGPR mRNA were higher in hepatic cancer and liver cells than in spleen, kidney, lung, muscle, heart and bowel tissues (P<0.01). The expression of ASGPR mRNA was higher in hepatic cancer cells than in any other tissue (P<0.01), and the highest expression in hepatic cancer cells was seen with GC/5-FU (P<0.01). With GC/5-FU the concentration of 5-FU in hepatic cancer cells was 8.69-, 23.35-, 79.96- and 85.15-times higher than in normal liver, kidney, heart and blood, respectively (Figure 4C).

### The Effect of GC/5-FU on the Tumor Mass and Survival in the Mouse Model

The tumor weights 15 days post-treatment were: 0.4361±0.1153 g in GC/5-FU group, 0.6142±0.1254 g in CS/5-FU group, 0.7932±0.1283 g in 5-FU group, 1.3989±0.2125 g in GC group and 1.5801±0.2821 g in control group (Figure 4D). The differences between the groups was statistically significant (P<0.01). Body weight between Day 1 and 10 decreased significantly more in the 5-FU group than in the control, GC, CS/5-FU and GC/5-FU groups (P<0.01). There was no difference in body weight changes in the GC/5-FU, CS/5-FU, GC and control groups (P>0.05), and no difference between the GC/5-FU and control group.

Kaplan-Meier analysis (Figure 4F) showed that the median survival was 16 days in the control group, with all the mice dying between Day 11 and 18. Median survival in the GC group was 17 days, with all mice dying between Days 10 and 20 (P>0.05). Mice treated with 5-FU had a median survival of 22 days, (mice died between Days 18 and 28), in the GS group the median survival was 29 days, (all mice died between Days 19 and 33). and in the GC/5-FU it was 35 days (all mice died between Days 20 and 41). Median survival with 5-FU CS/5-FU or GC/5-FU was significantly longer than with GC or control treatment (P<0.01).

### The Effect of GC/5-FU on the Cell Cycle, Proliferation and Apoptosis of H22 Cells

Flow cytometry was used to analyze the liver cancer samples harvested 15 days after beginning treatment. As shown in Figures 5A and 5B, the percentage of cells in the G0-G1 phases was significantly higher in the GC/5-FU and 5-FU groups (P<0.01), and the proliferation index (PI) was significantly lower, than in the GC and control groups (P<0.01).

In addition, the percentage of apoptotic cells was significantly higher in the GC/5-FU, CS/5-FU and 5-FU groups than in the control and GC groups (P<0.01). The percentage of apoptotic cells in the GC/5-FU group was higher than in the 5-FU group (P<0.01) and CS/5-FU group (P<0.05). However, there was no difference between 5-FU and CS/5-FU groups (P>0.05) (Figure 5B).

Apoptosis was detected on the tumor sections using the TUNEL assay. Tumor samples from the control and GC groups showed little apoptosis whereas the addition of 5-FU and CS/5-FU induced sporadic apoptosis and GC/5-FU induced a high degree of apoptosis with a clustered distribution (Figure 6A and 6C). The apoptosis index (AI) in tissue sections was significantly higher in the GC/5-FU, CS/5-FU and 5-FU groups than in the GC or control groups (P<0.01). It was also significantly higher in the GC/5-FU group than in the CS/5-FU or 5-FU groups (P<0.01). No difference in AI was found between the CS/5-FU and 5-FU groups (P>0.05).

### GC/5-FU Induced Hepatic Cancer Cell Apoptosis via Activating the p53 Pathway

To understand which pathway(s) mediated the GC/5-FU-induced apoptosis, we examined the expression of p53 at both protein and mRNA levels. The expression of p53 was higher in the 5-FU, CS/5-FU and GC/5-FU groups, than in the control and GC groups. The highest increase was seen in the GC/5-FU group (P<0.01, Figures 6B and 7A). The ratio of Bcl-2/Bax showed a decreasing tendency from control to GC to 5-FU to CS/5-FU to GC/5-FU groups (P<0.01, Figure 7D). Specifically, the Bcl-2/Bax ratio was significantly lower in the 5-FU, CS/5-FU and GC/5-FU groups than in the control or GC groups, with a lowest ratio observed with GC/5-FU group (P<0.01). GC/5-FU also significantly induced the expression of caspase-3 in tumor tissues at both protein and mRNA levels (P<0.01, Figure 6B and 7B), the expression of caspase-3 showed an increasing tendency from control to GC to 5-FU to CS/5-FU to GC/5-FU groups. As shown in Figure 7C, the expression of PARP-1 decreased from control to GC to 5-FU to CS/5-FU to GC/5-FU groups (P<0.01), with the most marked reduction being seen in the GC/5-FU group.

## Discussion

The utilization of nanotechnology and nano-materials in the pharmaceutical field has given rise to the development of drug-nanoparticle carrier-release systems Particles ranging from 0.1 nm to 100 nm are considered to be a nanoparticle [36]. Nanoparticle size is very important for drug delivery, as the spaces between the cells in various tissues differ. The aperture of vascular endothelial cells within most normal tissues is 2 nm, the aperture of the postcapillary venules is 6 nm, and the aperture of non-continuous tumor blood vessels ranges from 100 nm to 780 nm [37], [38]. The size of the nanoparticles used in this study (35.19 nm), was smaller than those used in most previously reported studies [39], allowing them to enter the spaces within tumor cells but restricting them from penetrating the normal tissues. Sweep electron microscope analysis showed that the particles were spherical in shape with a smooth surface and no adhesion between nanoparticles, which is consistent with previous reports [39], [40].

Bio-targeting, based on ligand–receptor interactions, is another important way of increasing drug delivery [41]. The linking of a targeting ligand to a nanoparticle carrier allows, multiple, specific bonds to be produced between the ligand and cancer cell receptors or tissues thereby enhancing the therapeutic activity. Asialoglycoprotein receptor (ASGPR) is found on the membrane of hepatocytes that face sinusoids, and has specificity for glycoproteins ending with a galactose moiety. ASGPR in cell membranes binds to target ligands to form a complex that enters cells by clathrin-mediated endocytosis [42]. Once inside the cell the ligands are released from ASGPR to allow receptor recycling at the cell membrane [43]. Rapid recycling of the internalized receptor is important for maintaining adequate receptor concentrations on liver parenchymal cell surfaces, which usually contain 100,000–500,000 binding sites per cell [43]. Once inside the cell the penultimate galactose glycoprotein residues are capped by terminal sialic acid moieties [44].Asialoglycoproteins are therefore, endogenous glycoproteins where the sialic acid has been removed by sialidase enzyme activity. The removal of sialic acid (N-acetylneuraminic acid) renders the now terminal galactose residues as recognition determinants for ASGPR [44]. In the present study, GC/-5-FU nanoparticles were synthesized and the binding of the galactose ligand with ASGPR was used to induce liver-targeted transfer.

In order to confirm sustained release and liver-specific targeting, we performed a series experiments on GC/5-FU. The in vitro release curve of GC/5-FU in simulated body fluid showed that the sustained release of the nanoparticle lasted from 1 to 8 days. This allowed the drug to be evenly distributed in the body, thereby increasing the half-life of GC/5-FU in the circulation and decreasing its toxic effects on normal tissues [45]. We also demonstrated that the in vitro cytotoxic effect GC/5-FU and CS/5-FU nanoparticles was attenuated on SW480 cells, as these cells did not express receptors for galactosyl (Figure 3A). This was may have resulted in lower concentrations of 5-FU being released from the nanoparticles at each time point. The cytotoxic effect of GC/5-FU on SMMC-7721 cells, which express a substantial level of galactosyl receptors, was significantly higher than that seen with 5-FU and CS/5-FU which have no galactosyl ligands. However, CS/5-FU attenuated the in vitro cytotoxic effect on SMMC-7721 cells. In the IC_50_ experiments, the dose-dependent cytotoxicity of 5-FU on SW480 and SMMC-7721 cells was effective during Days 1 to 6 but thereafter cytotoxicity plateaued. GC/5-FU acted as a sustained release agent for SW480 cells and CS/5-FU acted in the same manner for SMMC-7721 and SW480 cells allowing the drug to be continually released from Day 1 to 10. This resulted in the occurrence of time-dependent cytotoxic effects with these nanoparticles. Before Day 7 when the cumulative release of GC/5-FU and CS/5-FU was low, the IC_50_ for GC/5-FU-SW480, CS/5-FU-SW480, CS/5-FU-SMMC-7721 were higher than for 5-FU-SW480 and 5-FU-SMMC-7721. However, after Day 6 when the nanoparticles conferred high, effective concentrations of 5-FU, the IC_50_ values for GC/5-FU-SW480, CS/5-FU-SW480, CS/5-FU-SMMC-7721 were lower than for 5-FU-SW480 and 5-FU-SMMC-7721. The IC_50_ for GC/5-FU-SMMC-7721 (with SMMC-7721, which expresses galactosyl receptors), remained lower than that for 5-FU-SMMC-7721 throughout the 10-day period. It is proposed that the presence of a large number of ASGP receptors on the surface of hepatic cancer cells facilitated endocytosis of the GC/5-FU nanoparticles (which each contain the galactosyl ligands), thereby resulting in higher effective intracellular concentrations of 5-FU [46], [47]. Other cell types in the liver, such as Kupffer cells, endothelial cells and adipocytes, do not express galactosyl receptors. This means that the GC particle can be highly specific for those hepatic cells that do possess galactosyl receptors [25], [48].

Our in vivo experiments showed that there was a similar pattern of distribution of CS/5-FU and 5-FU alone between hepatic and non-hepatic tissues, whereas GC/5-FU significantly increased the 5-FU concentration in the hepatic tumor tissue, in comparison with normal liver tissue, kidney, heart tissue and blood. The tendency of differential drug distribution in various tissues is consistent with the expression of ASGPR mRNA which was shown by RT-PCR to be higher in hepatic cancer cells than in other tissues (Figure 4A). This supports previously published finding using GC as a carrier for gene delivery [27].The tumor weight of mice treated with GC/5-FU, CS/5-FU and 5-FU was significantly lower than in mice treated with GC or control with the lowest tumor weights being seen with GC/5-FU. Body weight decreased significantly compared with control in mice receiving 5-FU but not in any of the other treatment groups. This finding suggests that GC may prevent the gastrointestinal tract side effects associated with 5-FU that were responsible for weight loss. Median survival in the control, GC, 5-FU,CS/5-FU and GC/5-FU groups was 16, 17, 22, 29 and 35 days, respectively. These findings suggest that although GC alone does not affect tumor growth, its conjugation with 5-FU improves the cytotoxic effect of 5-FU by mediating its endocytosis 5-FU into liver cancer cells.

To determine the mechanism of effect of GC/5-FU nanoparticles on the hepatic cancer, we used flow cytometry and TUNEL assays to examine tumor cell apoptosis. The results showed that 5-FU, CS/5-FU and GC/5-FU enhanced apoptosis when compared to either control or GC, and that GC/5-FU increased the apoptosis index when compared to 5-FU and CS/5-FU. These findings suggest that GC improves the pro-apoptotic effect of 5-FU by promoting its entry into the cell. As shown in Figure 5A, 5-FU, CS/5-FU and GC/5-FU also increased the percentage of cells in the G0–G1 phases and lowered the PI. These results are consistent with previously reported research indicating that the cytotoxic effect of 5-FU,CS/5-FU and GC/5-FU on proliferating cells is mediated by causing the cell cycle to arrest in the G0–G1 phases [49], [50].

We also demonstrated that GC/5-FU, CS/5-FU and 5-FU induced p53 expression at both the protein and the RNA levels with the strongest induction of p53 being noted in the GC/5-FU group. Decreases in the Bcl-2/Bax ratio showed a similar pattern. Bax is a member of the Bcl-2 family that promotes apoptosis. Both Bax and Bcl-2 coexist in cells as dimers, and each suppresses the function of the other. Physiologically, both are present in cells in the same amounts, ensuring the normal cell growth. If Bcl-2 is overexpressed, the heterodimer Bcl-2/Bax is induced to suppress apoptosis, and if the level of Bax increases, the formation of Bax/Bax homodimer promotes apoptosis by antagonizing the anti-apoptotic effect of Bcl-2 [51]. Wild-type p53 induces Bax synthesis to mediate apoptosis, while mutant p53 can inhibit apoptosis leading to uncontrolled proliferation [52]. In our study GC/5-FU significantly enhanced caspase-3 expression, indicating that caspase-3 is activated and that it participates in the cell death pathway induced by GC/5-FU. Caspase-3 is one of the principle caspases found in apoptotic cells. Caspase-3 can be activated by cytochrome c in the cytosol. Cytochrome c is released from mitochondria into the cytosol under the control of Bax and Bcl-2. Therefore, the ratio of Bax and Bcl-2 determines the degree of activation of caspase-3 [53], [54]. We also demonstrated a tendency toward a decrease in PARP-1 expression with the most significant reduction being seen in the GC/5-FU group. Activation of PARP-1 following severe DNA damage results in depletion of cellular energy. In order to prevent the consumption of NAD+ and adenosine triphosphate, activated caspase-3 cleaves and inactivates PARP-1, which results in apoptosis [51]. Taken together the above results indicate that GC improves the apoptotic effect of 5-FU in hepatic cancer cells. The mechanism underlying GC/5-FU nanoparticle-induced apoptosis appears to involve induction of p53 expression at the protein and mRNA levels. The elevated p53 levels significantly lower the Bcl-2/Bax ratio which in turn promotes the release of cytochrome c from the mitochondria into the cytosol, leading to the activation of caspase-3. Upregulation of the caspase-3 gene and protein both contribute to the reduction in PARP-1 at both protein and mRNA levels, thus triggering apoptosis. Therefore, GC/5-FU-induced apoptosis is p53 dependent.

### Conclusion

Our results indicate that GC is a good carrier for nano-material, especially 5-FU. GC/5-FU nanoparticles have a sustained release effect, resulting in dose- and time-dependent targeted hepatic cell cytotoxicity. GC/5-FU nanoparticles inhibited tumor growth in the orthotropic liver cancer mouse model more markedly than 5-FU alone. The mechanism of action resulted from induction of G0–G1 arrest and apoptosis mediated by the p53 pathway.
